# Variance component estimations and mega‐environments for sweetpotato breeding in West Africa

**DOI:** 10.1002/csc2.20034

**Published:** 2020-01-27

**Authors:** Jolien Swanckaert, Daniel Akansake, Kwadwo Adofo, Kwabena Acheremu, Bert De Boeck, Raul Eyzaguirre, Wolfgang J. Grüneberg, Jan W. Low, Hugo Campos

**Affiliations:** ^1^ International Potato Center (CIP) PO Box 3785, Fumesua Kumasi Ghana; ^2^ CSIR‐Crops Research Institute (CSIR‐CRI) PO Box 3785, Fumesua Kumasi Ghana; ^3^ CSIR‐Savanna Agricultural Research Institute (CSIR‐SARI) P.O. Box 52 Tamale Ghana; ^4^ International Potato Center (CIP) Av. La Molina 1895 Lima Peru; ^5^ International Potato Center (CIP) Nairobi Kenya

## Abstract

The current study was aimed at identifying mega‐environments in Ghana and evaluating adaptability of superior sweetpotato [*Ipomoea batatas* (L.) Lam.] genotypes from a targeted breeding effort. Three sets of genotypes were evaluated in multi‐environment trials (MET). Twelve sweetpotato varieties were evaluated across nine environments representing the main agro‐ecological zones in Ghana. MET analysis was conducted using a stage‐wise approach with the genotype × environment (G × E) table of means used as a starting point to model the G × E interaction for sweetpotato yield. Emphasis was given to the genetic correlation matrix used in a second‐order factor analytic model that accommodates heterogeneity of genetic variances across environments. A genotype main effect and G × E interaction of storage root yield explained 82% of the variation in the first principal component, and visualized the genetic variances and discriminating power of each environment and the genetic correlation between the environments. Two mega‐environments, corresponding to northern and southern trial sites, were delineated. Six breeding lines selected from the south and eight breeding lines selected from the north were tested and compared to two common check clones at five locations in Ghana. A Finlay–Wilkinson stability analysis resulted in stable performances within the target mega‐environment from which the genotypes were selected, but predominantly without adaptation to the other region. Our results provide a strong rationale for running separate programs to allow for faster genetic progress in each of these two major West African mega‐environments by selecting for specific and broad adaptation.

**Abbreviations**
AEZ: agro‐ecological zone
AMMI: additive main effects and multiplicative interactions
BLUE: best linear unbiased estimator
BLUP: best linear unbiased predictor
CIP: International Potato Center
FAO: Food and Agriculture Organization of the United Nations
G × E: genotype by environment interaction
GGE: Genotypic main effects and genotype × environment interactions
IITA: International Institute of Tropical Agriculture
MET: multi‐environmental trial
RCBD: randomized complete block design
SPCSV: sweetpotato chlorotic stunt virus
SPFMV: sweetpotato feathery mottle virus
SPVD: sweetpotato virus disease
SSA: Sub‐Saharan Africa
SSP‐WA: Sweetpotato Support Platform for West Africa

## 1 INTRODUCTION

Sweetpotato [*Ipomoea batatas* (L.) Lam.] is cultivated across a wide range of agro‐ecological conditions, but storage root yields are low in many countries. Improving storage root yields can be achieved through genetic improvement and replacing old varieties by new ones, cultural practices such as timely planting, weed control, crop rotation and fertilizer input, and the use of pathogen‐tested clean planting material (Grüneberg et al., 2015). As most farmers in Sub‐Saharan Africa (SSA) do not have access to pathogen‐tested planting material, they rely on “apparently” healthy planting material obtained by negative selection which, in some cases, can be as effective as using pathogen‐tested planting material (Abidin, Akansake, Asare, Acheremu, & Carey, 2017). Good agricultural practices have been proven to boost sweetpotato productivity (Fuglie, Zhang, Salazar, & Walker, 1999; Lagnaoui, Alcázar, & Morales, 2000), but may not be accessible for smallholder farmers in SSA. The proportion of yield gap that can be met through improved varieties is generally estimated at 50%, but this is likely to be higher in West Africa where sweetpotato breeding is yet to be fully exploited (Andrade et al., 2009). The Food and Agricultural Organization of the United Nations (FAO) reports sweetpotato storage root yields across the world, with the lowest values (1.9 t ha^−1^ in 2016) in Ghana. However, actual yields are likely to be five times higher, as reported by the Ghanaian Ministry of Food and Agriculture, and similar to the values reported by FAO for its neighboring country, Burkina Faso (11.0 t ha^−1^; Ministry of Food and Agriculture, 2012).

Improving storage root yield through genetic improvement is a high priority in countries with low yields, although biotic and abiotic stresses can slow down genetic progress. An important biotic constraint in SSA is Sweetpotato Virus Disease (SPVD), especially in high virus‐pressure areas. The SPVD is caused by synergistic co‐infection by the whitefly‐transmitted crinivirus, Sweetpotato Chlorotic Stunt Virus (SPCSV), and the aphid‐transmitted potyvirus, Sweetpotato Feathery Mottle Virus (SPFMV; Clark et al., 2012; Gibson & Kreuze, 2015). Progress in breeding for SPVD resistance has been slow, most likely due to the hexaploid nature of sweetpotato and the reported recessive inheritance of resistance to SPCSV and SPFMV (Mwanga et al., 2002b; Mwanga, Yencho, & Moyer, 2002a). Furthermore, different strains of SPCSV are known to predominate in East and West Africa, and the implications of these differences for virus resistance breeding are not yet understood (Clark et al., 2012). Improved sweetpotato genotypes bred outside of Africa are usually unsuitable for SSA because in the majority of cases, they come from countries with lower virus pressure, and to a certain extent, better developed seed systems to manage virus pressure (Gibson & Kreuze, 2015). To be successful in West Africa, sweetpotato genotypes must have a good level of resistance to SPVD in the southern humid tropical agro‐ecological zones (AEZs) where vector populations and host reservoirs can remain high year‐round. They must also have a pronounced level of resistance to abiotic stresses in the drought‐prone northern savannah areas (Mwanga et al., 2002a).

Most released sweetpotato varieties in Ghana have been exotic introductions, coming from the International Institute of Tropical Agriculture's (IITA) sweetpotato breeding program (which phased out in 1987), East Africa (comprising a number of landraces), and Bangladesh. Sweetpotato introductions can bring about considerable genetic diversity because of the extreme heterozygosity of the crop (Grüneberg et al., 2015), but in countries with intensive production and established breeding programs, a large genetic diversity is already available for breeding (David et al., 2018). Expression of attributes and traits across environments can vary differentially by genotype, a phenomenon known as genotype × environment (G × E) interaction in multi‐environmental trial (MET) series. For any breeding program to be successful, a thorough understanding of G × E patterns in the target population of environments is essential. In cases where variance component estimations due to G × E ($\sigma_{G\times E}^{2}$) in METs are larger than variance component estimations due to genotypes ($\sigma_{G}^{2}$), an analysis of G × E interactions should be undertaken (Fox, Crossa, & Romagosa, 1997). For sweetpotato storage root yield, the main commercial trait in this crop, large G × E interaction have been reported across environments in Kenya and Uganda (Grüneberg, Abidin, Ndolo, Pereira, & Hermann, 2004; Tumwegamire et al., 2016), over diverse environments in Peru (Grüneberg, Manrique, Zhang, & Hermann, 2005), in South Africa (Adebola, Shegro, Laurie, Zulu, & Pillay, 2013), and in Ethiopia (Gurmu, Hussein, & Laing, 2017). These studies highlight the importance of breeding regionally adapted material and testing new genotypes under conditions similar to the targeted population of environments. The target environment should be well‐defined as a portion of the growing region with a fairly homogeneous environment that causes genotypes to perform similarly (Gauch & Zobel, 1997). The technique of subdividing growing regions in mega‐environments was first introduced by researchers at the International Maize and Wheat Improvement Center (CIMMYT), working with wheat (*Triticum aestivum*; Rajaram, 1994). The strategy of defining mega‐environments allows a breeder to take specifically adapted genotypes into account and to identify locations suitable for field tests and selection.

It is hypothesized that AEZs in southern and northern Ghana are contrasting, and exhibit a pronounced genotype × environment (G × E; $\sigma_{G\times E}^{2}>\sigma_{G}^{2}$) for storage root yield. It is also hypothesized that northern and southern environments in Ghana are representative of similar environment across the sub‐region which is broadly characterized by longer rainy seasons in the southern zones and longer dry seasons in the northern savanna zones. Information on sweetpotato G × E in Ghana is limited, and a better understanding of sweetpotato G × E in Ghana is needed to make more informed choices in sweetpotato breeding for both Ghana and West Africa.

The International Potato Center's (CIP) sweetpotato breeding efforts in SSA are organized at a sub‐regional level through support platforms to provide a foundation for pre‐breeding and long term population improvement and development of user‐preferred varieties (Andrade et al., 2009). The Sweetpotato Support Platform for West Africa (SSP‐WA) works closely with the Ghana National Agricultural Research and Extension Systems (NARES) to support breeding efforts in Ghana and across the region. This study had the following objectives: (i) to determine the magnitude of G × E interaction for storage root yield in multi‐environmental trials (METs) across AEZs in Ghana, (ii) to define mega‐environments for sweetpotato breeding in Ghana, and (iii) to identify superior breeding lines for each mega‐environment.

## 2 MATERIALS AND METHODS

Three different datasets were used to address the three objectives. A total of 26 genotypes varying in origin and root flesh color (Table 1) contributed to the three datasets. The first dataset consisted of 10 germplasm introductions and landraces, most either released or currently important varieties in Ghana. The second dataset represented six advanced clones from the sweetpotato breeding program at SSP‐WA selected in the South, while the third represented eight advanced clones selected in the North. Two check clones (CRI‐Apomuden and CRI‐Ligri) were common to all trials.

**Table 1:** Description of sweetpotato genotypes used for the genotype × environment interaction analysis

Name	DOI^a^	Origin (Pedigree)	Root flesh color	Dataset
CRI‐Apomuden	10.18730/MYC47	Bangladesh (Kamala Sundari; CIP440293)	Orange	1, 2, and 3
Blue Blue	10.18730/MYC7A	Ghana (Landrace)	White	1
CRI‐Bohye	10.18730/MYC8B	CIP199062.1 (SPV 78.001 × PC99_2)	Light orange	1
CRI‐Dadanyuie	10.18730/MYCCF	KEMB 37 (KSP20/TIS 2534)	Cream	1
CRI‐Faara	10.18730/MYCDG	IITA‐TIS 3017	Cream	1
King‐J	10.18730/MYCFJ	Nigeria (released variety)	Light orange	1
CRI‐Ligri	10.18730/MYCGK	Cuba (Cemsa 74–228; CIP400004)	Cream	1, 2, and 3
Mother's Delight	10.18730/MYCHM	Nigeria (introduced clone, identity unknown)	Orange	1
CRI‐Ogyefo	10.18730/MYCKP	Rwanda (Mugande)	White	1
CRI‐Okumkom	10.18730/MYCMQ	IITA‐TIS 8266	Cream	1
CRI‐Otoo	10.18730/MYCNR	Burundi (Mogamba; CIP440034)	Cream	1
CRI‐Sauti	10.18730/MYCPS	Uganda (Tanzania; CIP440166)	Yellow	1
PGA14008–15	10.18730/MYCRV	Ghana (CRI‐Otoo × OP)	Yellow	2
PGA14008–22	10.18730/MYCSW	Ghana (CRI‐Otoo × OP)	Yellow	2
PGA14011–13	10.18730/MYCVY	Ghana (CRI‐Apomuden × OP)	Cream	2
PGA14171–1	10.18730/MYCX*	Ghana (CIP199062.1/2 × CRI‐Faara)	Pale orange	2
PGA14229–2	10.18730/MYCY∼	Ghana (CRI‐Faara × SPK004/6)	Pale orange	2
PGA14351–36	10.18730/MYCZ$	Ghana (PGA12160–72 × PGA12151–75)	Dark cream	2
PGA14011–24	10.18730/S8711	Ghana (CRI‐Apomuden × OP)	Yellow	3
PGN16021–39	10.18730/S8722	Ghana (CIP105058.2 × CIP440004)	Orange	3
PGN16024–27	10.18730/S8733	Ghana (CIP105212.1 × CIP440012)	Orange	3
PGN16024–28	10.18730/S8744	Ghana (CIP105212.1 × CIP440012)	Orange	3
PGN16030–30	10.18730/S8755	Ghana (CIP194581.4 × CIP440004)	Pale orange	3
PGN16092–6	10.18730/S8766	Ghana (CIP105083.1 × CIP440004)	Cream	3
PGN16130–4	10.18730/S8777	Ghana (Diedi × CRI‐Otoo)	Purple	3
PGN16203–18	10.18730/S8788	Ghana (CIP194556.56 × CIP440004)	Orange	3

a DOI, Digital Object Identifier.

**Figure 1:**
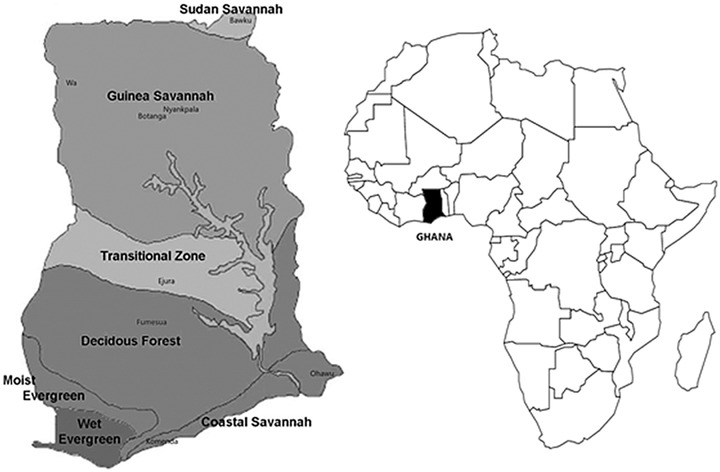
Map of Ghana with indication of trial sites and agro‐ecological zones (modified from FAO, 2005)

Each dataset was evaluated at southern and northern locations in Ghana (Table 2; Figure 1). Soil groups in Africa as classified by the FAO, identify the southern locations as Acrisols whereas the northern locations are Lixisols (Grüneberg et al., 2019). All trials were planted in the main rainy season, and harvest was conducted between 4–6 mo after planting (Table 2). The annual average rainfall ranges from 950–1200 mm. Good agricultural practices were used, with appropriate fertilization N/P/K rates (40:40:70 kg ha^−1^) and regular weeding with sweetpotato grown in rotation with other crops.

**Table 2:** Description of trial sites used in this study

Location^a^	Agro‐ecological zone	Latitude	Longitude	masl^b^	Dataset	Year	Soil type	Planting date	Harvest date
Ejura	Transitional	7°23′5″ N	1°21′32″ W	235	1	2018	Loamy sand	9 July 2018	23 Nov. 2018
Fumesua	Forest	6°42′39″ N	1°31′2″ W	289	1	2017	Loamy sand	8 June 2017	23 Oct. 2017
					1	2018	Silty loam	27 Sept. 2017	27 Jan. 2018
					2	2016	Loamy sand	17 May 2016	3 Oct. 2016
					3	2018	Loamy sand	7 May 2018	15 Nov. 2018
Ohawu	Coastal Savannah	5°3′20″ N	1°29′39″ W	9	1	2017	Sand	15 May 2017	12 Oct. 2017
					2	2016	Sand	1 June 2016	18 Oct. 2016
					3	2018	Sand	14 June 2018	15 Oct. 2018
Komenda	Coastal Savannah	6°7′52″ N	0°53′52″ E	35	1	2017	Loamy sand	15 June 2017	16 Oct. 2017
Nyankpala	Guinea Savannah	9°24′0″ N	0°59′0″ W	194	1	2017	Silty loam	27 Sept. 2017	27 Jan. 2018
					2 and 3	2017	Sandy loam	13 July 2017	25 Nov. 2017
Botanga	Guinea Savannah	9°36′12″ N	1°01′54″ W	111	1	2017	Sandy loam	30 Aug. 2017	6 Jan. 2018
					1	2017	Clayey loam	12 Dec. 2017	24 Apr. 2018
Tono	Guinea Savannah	10°3′15″ N	1°06′57″ W	233	1	2017	Laterite	5 Aug. 2017	2 Dec. 2017
Wa	Guinea Savannah	10°3′36″ N	2°30′36″ W	297	2 and 3	2017	Sand	17 July 2017	13 Nov. 2017
Bawku	Sudan Savannah	11°3′15″ N	0°14′19″ W	169	2 and 3	2017	Loamy sand	8 July 2017	2 Nov. 2017

a Southern environments: Ejura, Fumesua, Ohawu, and Komenda; Northern environments: Nyankpala, Botanga, Tono, Wa, and Bawku.

b masl, meters above sea level.

Trials were planted using randomized complete block design (RCBD) with two replicates, and plots consisting of four rows each 5 m, with a planting distance of 0.3 m between plants and 1 m between rows. The central 10 m^2^ of every plot were harvested roughly 4 mo after planting. After harvesting all the available plants, all storage roots in a plot were counted to calculate number of roots per plant. Roots were separated into noncommercial (<100 g) and commercial (≥100 g) sizes, and yield was recorded for both fractions. Total storage root yield was expressed as tons per ha. Total biomass (fresh roots + vines) was weighed per plot and expressed as tons per ha. Harvest index (total root yield divided by biomass yield) was reported as a percentage. The SPVD was scored 8 wk after planting from 1–9, with 1 having no symptoms and 9 having severe symptoms. Phenotyping traits are fully described in Grüneberg et al. (2019).

Statistical analysis was based on linear mixed models and was performed using R (version 3.3.1) and ASReml. Using Dataset 1, variance components for the nine environments were estimated by fitting a simple mixed model to the single RCBD trial data, considering the genotype main effects as random. Broad‐sense heritabilities (H^2^) were estimated as the proportion of estimated genotypic variance over the sum of estimated genotypic variance and estimated variance of the error, divided by the number of replicates.

For the MET analysis, a stage‐wise approach was used as elaborated in Piepho, Möhring, Schulz‐Streeck, and Ogutu (2012) and van Eeuwijk, Bustos‐Korts, and Malosetti (2016). In the first stage, the same nine models as above are fitted but considering the genotype effect as fixed, to obtain the Best Linear Unbiased Estimators (BLUEs) of the genotypes for each individual environment. This leads to a G × E table of BLUE means, which is used as a starting point to model the G × E interaction as described in Malosetti, Ribaut, and van Eeuwijk (2013) in the second stage. To predict the performance of a genotype in a given environment, Piepho (1994) and Piepho, Möhring, Melchinger, and Büchse (2008) show that Best Linear Unbiased Predictor (BLUP) often outperforms other procedures in terms of predictive accuracy. To this end, a mixed model considering genotype as a random effect is fitted in the second stage by residual maximum likelihood (REML). In the presence of G × E, the more realistic models often allow for heterogeneity of genetic variances and covariances across environments (Malosetti et al., 2013). The best fitting model was chosen by Akaike's information criterion (AIC) and took the following form: $\mu_{ij}=\mu +G_{i}+E_{j}+E_{ij}$ where *μ* is a fixed intercept, *E_j_* is a fixed environment effect, and $G_{i}∼N\left(0,\sum G\right)$ and $E_{ij}∼N\left(0,\sigma_{\varepsilon }^{2}\right)$ are normally distributed and independent random effects. The variance‐covariance matrix ∑G of the random genetic effect *G_i_* is parametrized using the second‐order factor analytic model that accommodates heterogeneity of genetic variances and genetic covariances across environments in a parsimonious manner (Piepho, 1998). The correlation matrix corresponding to this estimated 9 by 9 variance‐covariance matrix ∑G for storage root yield is given in Table 4.

Since we are interested in determining mega‐environments in Ghana, we made a graphical representation of the genotype main effects and the G × E effects (GGE), considering the following fixed GGE model: $\mu_{ij}=\mu +E_{j}+b_{i1}z_{j1}+b_{i2}z_{j2}+\varepsilon_{ij}$in the second stage. The model describes the response variable, that is, the BLUE of genotype *i* in environment *j*, *μ_ij_*, as the result of the common fixed intercept term *μ*, a fixed environmental main effect corresponding to environment *j*, *E_j_*, plus two multiplicative terms, *b_i1_z_j1_* and *b_i2_z_j2_*, approximating (G + G × E), and finally the random term, *ε_ij_*, representing the error term. Because we are working on a two‐way table of BLUEs, we cannot straightforwardly separate G × E from error. More details can be found in Yang, Crossa, Cornelius, and Burgueño (2009) and Malosetti et al. (2013). The results of the GGE analysis for storage root yield are presented in a biplot graph capturing genotypes and environment variation and covariation of the trials.

A similar linear first stage model was applied to each environment in the second and third dataset, considering genotype as a fixed effect and resulting in a G × E table of the estimated means. These means were then used to calculate the Finlay–Wilkinson model (Finlay & Wilkinson, 1963) with a single regression line on the environmental quality in the model $\mu_{ij}=\mu +G_{i}+E_{j}+b_{i}E_{j}+\varepsilon_{ij}$. The slope *b_i_* captures the environmental sensitivity or adaptability (Malosetti et al., 2013).

## 3 RESULTS

### 3.1 First dataset

**Figure 2:**
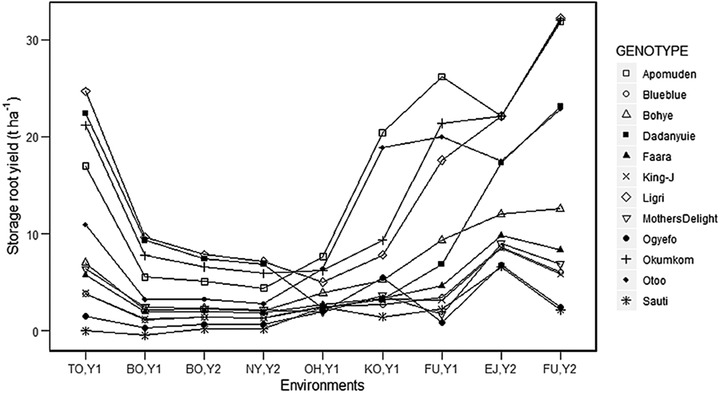
Storage root yield of twelve clones of sweetpotato: Multi‐environmental trial analysis done with a stage‐wize approach allowing for heterogeneous genetic variances and correlations between nine environments (BO, Botanga; EJ, Ejura; FU, Fumesua; KO, Komenda; NY, Nyankpala; OH, Ohawu; TO, Tono; Y1, Year 1, 2017; Y2, Year 2, 2018)

The nine environments of the first dataset expressed a large variability. Therefore, the estimated means were calculated for each environment separately following a two‐stage analysis strategy and these estimated means served as the basis for the G × E study. Estimated means for storage root yield ranged from 3–15.5 t ha^−1^ across environments, when averaging out the genotypes in the first dataset (Table 3). The $\sigma_{G}^{2}$ variance component had large differences among locations for all traits. The magnitude of the variance component $\sigma_{G}^{2}$ resulted in high heritabilities (H^2^), which were greater than .75 for most traits and locations. A graphical presentation of storage root yield BLUPs shows a clear crossover between genotype estimates across environments (Figure 2). As expected, low‐yielding locations expressed a lower $\sigma_{G}^{2}$ component than higher‐yielding locations, visualized on Figure 2 for storage root yield.

**Table 3:** Means, coefficient of variation, variance components and heritability for observed traits at nine locations (obtained from single trial analysis with random genotype effect)

		Botanga	Fumesua	Komenda	Ohawu	Tono	Botanga	Ejura	Fumesua	Nyankpala
			
		2017					2018			
Storage root yield, t ha^−1^	mean	3.7	9.8	7.1	3.8	10.4	3.4	13.6	15.5	3.1
	CV^a^, %	57.8	24.7	62.7	36.1	25.4	40.3	33.8	32.6	67.2
	$\sigma_{G}^{2}$	13.1	82.4	35.5	6.8	76.3	12.5	34.4	140.2	6.8
	$\sigma_{\mathrm{Error}}^{2}$	4.6	5.8	19.7	1.9	7.0	1.8	21.0	25.4	4.3
	H^2^	.85	.97	.78	.88	.96	.93	.83	.94	.76
Number of roots	mean	1.2	2.2	2.6	1.7	3.2	1.5	2.5	3.0	1.3
per plant	CV, %	32.1	26.4	23.9	30.4	26.6	55.5	25.7	22.2	45.0
	$\sigma_{G}^{2}$	1.0	2.1	1.8	.4	3.4	3.2	1.5	2.6	.6
	$\sigma_{\mathrm{Error}}^{2}$	.1	.3	.4	.3	.7	.7	.4	.5	.4
	H^2^	.93	.93	.9	.75	.91	.9	.91	.95	.75
Harvest index, %	mean	32.3	27.6	26.3	36.2	38.7	31.7	94.4	38.6	42.3
	CV, %	43.3	28.3	38.0	15.9	17.9	44.6	3.3	24.6	21.7
	$\sigma_{G}^{2}$	550.2	267.8	167.3	242.7	401.7	605.7	1.8	313.3	505.7
	$\sigma_{\mathrm{Error}}^{2}$	195.7	61.2	99.6	33.1	48.0	201.2	9.5	87.7	83.8
	H^2^	.85	.90	.77	.94	.94	.86	.36	.91	.92
Biomass yield, t ha^−1^	mean	10.9	29.4	24.2	11.1	22.8	9.1	14.3	34.6	6.1
	CV, %	120.7	19.2	51.5	29.1	19.5	38.7	32.4	29.6	60.4
	σ^2^_G_	11.9	199.3	47.2	53.5	110.8	21.3	38.6	251.8	8.8
	$\sigma_{\mathrm{Error}}^{2}$	174.3	32.0	155.9	10.4	19.7	11.8	21.6	110.2	13.6
	H^2^	.12	.93	.38	.91	.92	.78	.84	.87	.56
SPVD (1 = no symptoms;	mean	2.00	4.00	4.00	3.92	1.67	1.21	–	3.66	1.46
9 = severe symptoms)	CV, %	39.4	10.2	16.9	16.0	33.0	39.1	–	21.2	38.5
	$\sigma_{G}^{2}$	2.01	1.19	.55	.20	.64	0	–	2.29	.32
	$\sigma_{\mathrm{Error}}^{2}$	.62	.17	.45	.39	.30	.18	–	.61	.31
	H^2^	.87	.93	.71	.51	.81	0	–	.92	.67

a CV, coefficient of variance; $\sigma_{G}^{2}$, variance component estimations due to genotypes; H^2^, broad‐sense heritability; $\sigma_{\mathrm{Error}}^{2}$ variance component estimations due to error; SPVD, Sweetpotato Virus Disease.

**Figure 3:**
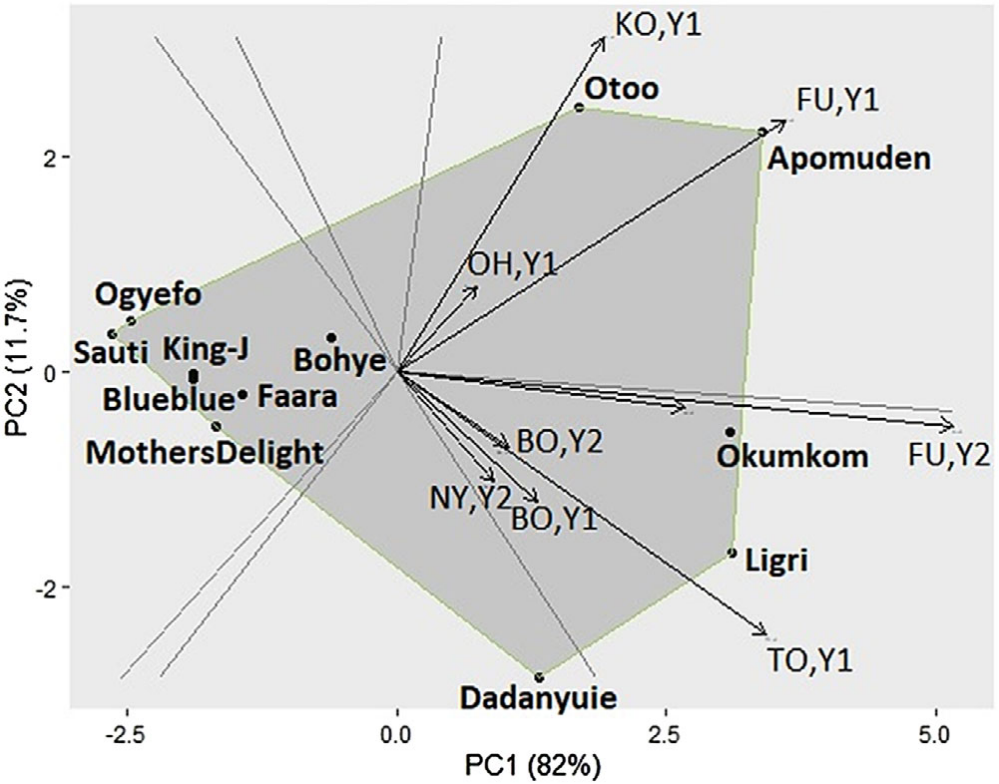
The genotype main effect and genotype × environment interaction plot of twelve sweetpotato genotypes evaluated for storage root yield in nine environments (BO, Botanga; EJ, Ejura; FU, Fumesua; KO, Komenda; NY, Nyankpala; OH, Ohawu; TO, Tono; Y1, Year 1, 2017; Y2, Year 2, 2018; PC1, Principle Component 1; PC2, Principle Component 2)

The genetic correlations between the nine environments, given in Table 4, are also reflected in the GGE plot (Figure 3; Table 5). Northern locations (Botanga, Nyankpala, and Tono) were highly correlated, indicated by the small angle between their environmental vectors. The southern locations grouped according to year. The GGE plot visualizes the genotypic adaptation, with the genotypes on the corners of the polygon most adapted to the environment pointing in that direction. The length of the environmental vectors gives an indication of the genetic variances in each trial with Botanga, Nyankpala, and Ohawu having the smallest genetic variance, as also shown in Figure 2. That means that these locations had the least discriminating power.

**Table 4:** Genetic correlation matrix based on storage root yield in nine environments used in dataset1 (BO, Botanga; EJ, Ejura; FU, Fumesua; KO, Komenda; NY, Nyankpala; OH, Ohawu; TO, Tono; Y1, Year 1, 2017; Y2, Year 2, 2018)

	TO,Y1	BO,Y1	BO,Y2	NY,Y2	OH,Y1	KO,Y1	FU,Y1	EJ,Y2	FU,Y2
TO,Y1	1	.9918	.9976	.9937	.5627	.3912	.6963	.9265	.9315
BO,Y1	.9918	1	.9983	.9999	.4524	.2870	.5989	.8708	.8774
BO,Y2	.9976	.9983	1	.9991	.5040	.3355	.6449	.8982	.9041
NY,Y2	.9937	.9999	.9991	1	.4662	.2999	.6112	.8783	.8847
OH,Y1	.5627	.4524	.5040	.4662	1	.8729	.9851	.8324	.8248
KO,Y1	.3912	.2870	.3355	.2999	.8729	1	.8392	.6596	.6516
FU,Y1	.6963	.5989	.6449	.6112	.9851	.8392	1	.9152	.9096
EJ,Y2	.9265	.8708	.8982	.8783	.8324	.6596	.9152	1	.9999
FU,Y2	.9315	.8774	.9041	.8847	.8248	.6516	.9096	.9999	1

**Table 5:** Analysis of variance for genotype main effect and genotype × environment interaction of storage root yield for 12 sweetpotato genotypes in nine environments

					Explained variation
Source of variation	df^a^	SS	MS	*P*‐value	%
Environment	8	2141.7	267.7	<.001	
Interactions	99	4919.6	49.7		
PC1	18	4032.1	224.0	<.001	82.0
PC2	16	573.8	35.9	<.001	11.7
PC3	14	148.7	10.6	<.001	3.0
PC4	12	65.2	5.4	.038	1.3
Residuals	39	99.7	2.6		

a df, degrees of freedom; SS, sum of squares; MS, mean square; PC1–4, Principle Components 1, 2, 3, and 4, respectively.

### 3.2 Second and third dataset

Trait estimates of advanced southern‐selected breeding clones were compared with the estimates of advanced breeding clones from successive northern breeding trials in Table 6. Storage root yield was consistently higher for the northern‐selected advanced clones compared to the southern‐selected advanced clones, evaluated across the country. Other traits were comparable between the two groups of genotypes. Check clones CRI‐Apomuden and CRI‐Ligri were common to both datasets, but their performance varied between years.

**Table 6:** Mean estimates (x̄_i_) and variance components (σ_i_^2^) using a mixed model for two check clones^a^, six southern advanced clones (dataset 2) and eight northern advanced clones (dataset 3)

	Storage root yield				Biomass yield		Harvest index	
	t ha^−1^		Number of roots per plant		t ha^−1^		%	
Genotypes	x̄_i_	σ_i_^2^	x̄_i_	σ_i_^2^	x̄_i_	σ_i_^2^	x̄_i_	σ_i_^2^
**Dataset 2: Southern clones evaluated in the South in 2016 and in the North in 2017**								
CRI‐Apomuden^a^	17.1	14.5	3.8	1.3	24.4	20	68.9	8.8
CRI‐Ligri^a^	12.5	7.5	2.6	1.1	20.2	11.7	61.8	5.2
PGA14008–15	9.2	9.5	2.0	1.2	18.5	15.8	47.3	12.2
PGA14008–22	14.1	7.7	3.0	1.3	20.6	12.3	73.0	8.3
PGA14011–13	21.5	11.0	4.0	1.4	38.1	20.6	58.9	14.0
PGA14171–1	10.2	9.8	4.2	1.8	30.1	23.5	38.1	17.1
PGA14229–2	12.8	12.2	3.0	1.8	27.5	34.3	54.9	12.8
PGA14351–36	15.4	13.0	2.6	.9	36.4	34.3	45.8	10.4
LSD (.05)^b^	7.53		1.17		19.20		11.16	
Levene Test		NS		NS		NS		NS
**Dataset 3: Northern clones evaluated in the North in 2017 and in the South in 2018**								
CRI‐Apomuden^a^	14.4	6.9	3.8	.9	22.6	8.9	63.1	14.5
CRI‐Ligri^a^	15.2	4	2.4	1.3	28.4	7.4	53.8	16.3
PGA14011–24	18.4	8.6	4.4	1.4	32.8	10.2	56.2	15.2
PGN16021–39	19.6	4.0	3.5	.8	37.0	18.5	63.1	26.6
PGN16024–27	24.4	9.3	3.8	.8	53.6	31.3	51.2	14.6
PGN16024–28	20.3	9.8	3.0	.9	41.2	9.3	51.0	24.0
PGN16030–30	21.8	5.6	4.8	1.2	35.9	12.1	64.6	12.5
PGN16092–6	19.1	13.9	2.8	1.0	26.9	16.4	63.2	20.6
PGN16130–4	14.8	7.1	3.6	.9	22.7	16.9	71.9	15.1
PGN16203–18	16.2	9.6	2.9	1.6	24.8	12.3	61.8	19.6
LSD (.05)	8.16		1.05		16.23		11.05	
Levene Test		NS		NS		NS		NS

a check clones.

b LSD, least significant difference; NS, nonsignificant.

**Figure 4:**
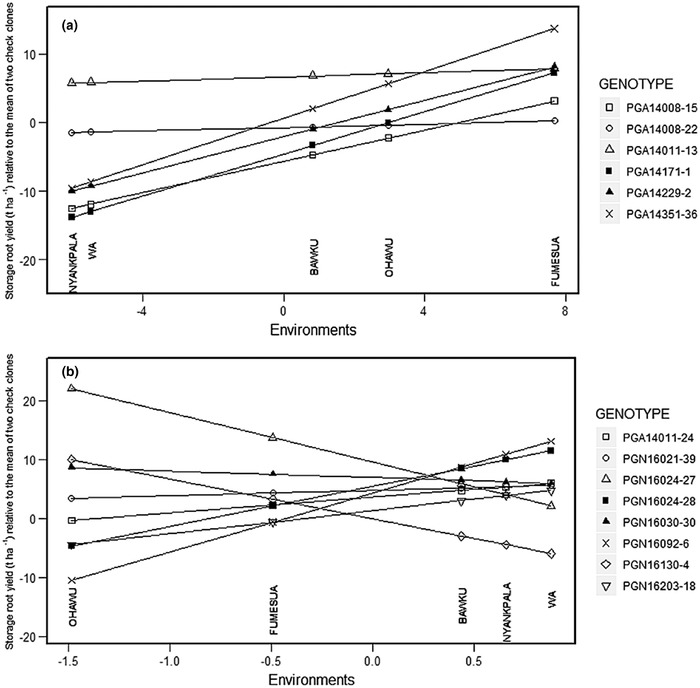
Finlay–Wilkinson stability plot for storage root yield (t ha^−1^) of (a) six advanced southern clones and (b) eight advanced northern clones evaluated in Southern sites (Fumesua and Ohawu) and in Northern sites (Nyankpala, Bawku, and Wa), relative to mean of the two check clones, CRI‐Apomuden and CRI‐Ligri

The Finlay–Wilkinson stability model used the relative root yield of each genotype compared to the mean of the two check clones. The subdivision of G × E sum of squares (Table 7) showed that the regression explained about three‐fourths of the total G × E interactions for storage root yield. The performance of the advanced clones is expected to be higher than the mean of the check clones in the region of selection. Indeed, in Figure 4a, most genotypes performed better than the mean of the two checks when they were evaluated in the Southern locations (Fumesua and Ohawu).

**Table 7:** Analysis of variance with subdivision of genotype × environment interactions using regression analysis on storage root yield genotype × environment means following the Finlay–Wilkinson model

Dataset 2: southern advanced clones					Dataset 3: northern advanced clones				
Effect	df^a^	SS	MS	*P*‐value	Effect	df	SS	MS	*P*‐value
Genotype	5	486	97.3	<.001	Genotype	7	319	45.6	.052
Environment	4	806	201.4	<.001	Environment	4	97	24.3	.299
Regression	5	322	64.3	<.001	Regression	7	1005	143.6	<.001
Error	15	111	7.4		Error	21	389	18.5	

df, degrees of freedom; SS, sum of squares; MS, mean square.

This result is in line with the expectation because these southern clones have survived earlier selection steps performed in the South, mainly based on superior storage root yield, high number of roots per plant, and low incidence of SPVD. Evaluating the southern clones in the northern locations (Bawku, Nyankpala, and Wa) revealed only one genotype better than the mean of the checks. The genotype PGA14011–13 can therefore be seen as a broadly adapted genotype, associated with a small slope. All other southern clones fall below the reference line, meaning that their performance was below the mean of the two check clones. Evaluation of northern clones (Figure 4b) showed a good performance in the northern locations, except for PGN16130–4 which has been selected for its particular purple flesh color. Four northern clones had a positive performance (better than the mean of the two check clones) in both southern locations.

## 4 DISCUSSION

Total storage root yield across all three datasets ranged from 3–22 t ha^−1^. The high yields corresponded with the yields found by Grüneberg et al. (2004) across 12 East‐African environments, and Abidin et al. (2017) who studied sweetpotato planting material in northern Ghana using the same check genotypes. Low yields were found in the first dataset where all genotypes were introductions or landraces. Storage root yields of CRI‐Apomuden and CRI‐Ligri, used as check clones, were consistent across the three datasets. Storage root yield was reported in this study as the sum of commercial and noncommercial root weight but the need to measure both commercial and noncommercial root weight is debatable (Grüneberg et al., 2015). Harvest index can be a predictor of yield stability as evidenced in wheat and maize (*Zea mays*; Bolaños & Edmeades, 1993). Intuitively, genotypes with a high harvest index are preferred, although farmers growing sweetpotato in low‐yielding environments do not desire a very high harvest index because aboveground biomass is needed as planting material, or in some cases for use as vegetable or fodder.

Screening genotypes in multi‐environmental trials requires an understanding of the G × E effects and yield stability across the environments. A two‐stage mixed model analysis handled the variation within trials in a first step during the calculating of the G × E prediction means. In the second step, the prediction means from all trials— each with a specific genotypic variation— were combined to fit the GGE model. The GGE model is preferred over an additive main effects and multiplicative interaction (AMMI) model because it does not partition the genotype main effect G and the G × E effect, GE (Yan, Kang, Ma, Woods, & Cornelius, 2007). The authors point out that the effect G is always specific to the environment in which it is estimated. Moreover, GE becomes G when environments are subdivided into mega‐environments. Sweetpotato root yields are highly affected by environment, also indicated by previous G × E studies (Adebola et al., 2013; Grüneberg et al., 2004, 2005; Gurmu et al., 2017; Tumwegamire et al., 2016). In agreement with a study from Papua New Guinea (Wera et al., 2018), the calculated mega‐environments were formed relating to the distinctiveness of major AEZ in which the trials were conducted. Furthermore, broad and specific adaptation of breeding clones from a targeted breeding effort in each mega‐environment was confirmed after field evaluation in both the targeted and nontargeted breeding environment.

The division between South and North Ghana makes sense for agricultural reasons. Sweetpotato production in the southern parts of Ghana suffers from a high virus pressure compared to the northern parts of Ghana, due to the longer rainy season in the South which supports virus vector and host populations, while the long dry season in the North tends to reduce virus pressure. Differences in sweetpotato performance between the North and South may also be explained by differences in soil classification of the two mega‐environments. The mega‐environments seem to coincide with the tropical forest environments in the South, and savannahs in the North which extend across West Africa. However, more studies will help to determine the representativeness of our mega‐environments to the rest of the West African region.

Subdividing a growing region implies more work for the plant breeder and seed producers, but it also implies faster progress and higher yields (Gauch & Zobel, 1997). One could ask whether there is a need for highly resistant varieties in the northern environments, with lower virus pressure, in which case, we have demonstrated that more progress can be made by conducting separate breeding programs for low and high virus‐pressure environments, coinciding with the two mega‐environments. As sweetpotato in the North was generally lower yielding, we expect that the farmers in such environments would be the main beneficiaries from a separate breeding program, which will allow us to select strongly for yield and preferred quality attributes, without the constraint presented by the need to select for high levels of virus resistance.

This study uses one set of check clones for both the South and North, but the need for specific check clones for each mega‐environment may arise. The commercial genotype CRI‐Apomuden is a highly demanded released variety in Ghana because of its high content of β‐carotene (correlated with the orange flesh color) and high yield. The sweetpotato genotype CRI‐Ligri, also known as Cemsa‐74–228, originated from Cuba and has been officially released in Ghana. CRI‐Ligri is also used as a globally adapted check genotype in CIP's sweetpotato breeding program, with sites in Ghana, Mozambique, Uganda, and Peru. CRI‐Ligri had a high total root yield in East‐Africa (33 t ha^−1^ reported by Grüneberg et al., 2004) and Abidin et al. (2017) reported a total root yield of 15.4 t ha^−1^ of CRI‐Ligri grown in northern regions in Ghana, which is in line with our results.

Despite the differences in soil type and soil fertility, each trial in both North and South Ghana received the same amount of fertilizers to keep the differentiation between environments. Differences among genotypes were larger in high‐yielding environments than in low‐yielding environments, observed in this study and confirmed by Grüneberg et al. (2005), which makes selection preferred in high‐yielding environments. Indeed, the high‐yielding environments are the discriminating environments in the GGE biplot. On the other hand, a challenging environment (e.g., high virus pressure) creates high variation in the performances which helps selecting the materials that stand out. Both favorable and less‐favorable environments should be used in the early yield testing stages of a sweetpotato breeding program to select for genotypes with wide adaptation. Breeding for high‐yielding genotypes with wide adaptation is possible but it is not a guarantee that these genotypes will outperform the genotypes with specific adaptation to marginal environments when grown in these marginal environments (Grüneberg et al., 2005). Therefore, check clones in a breeding program should include widely adapted (with low contribution to G × E) and specifically adapted genotypes that perform well in the targeted environment.

Until 2016, sweetpotato breeding in Ghana has been largely conducted in the South, with national releases made after limited testing in the North, resulting in some not adapted to the northern savannah environments at all. Our study confirmed that targeted breeding efforts in northern Ghana resulted in specifically adapted genotypes for the northern conditions. On the other hand, selection in the south is needed to find specifically adapted genotypes for the southern conditions. Both selection procedures have led to broadly adapted genotypes for all locations in Ghana.

In conclusion, improving sweetpotato root yield through breeding in Ghana can be achieved by selecting for broadly or specifically adapted genotypes. We have characterized the trial sites used for breeding purposes in Ghana and found a clear difference between northern and southern locations. Therefore, our results support the breeding strategy of selecting superior genotypes in both regions independently, while routinely evaluating for broad adaptation across northern and southern sites as part of the screening process. This will enable faster genetic progress in these two major West African mega‐environments.

## CONFLICT OF INTEREST

The authors declare that there is no conflict of interest.

## AUTHOR CONTRIBUTIONS

Results in this study were obtained at the Sweetpotato Support Platform for West Africa, where Jolien Swanckaert was based. She designed and managed different aspects of the work and wrote the manuscript with contributions from other authors. Daniel Akansake is sweetpotato breeder at CIP in Ghana. Kwadwo Adofo and Kwabena Acheremu are sweetpotato breeders based at CRI and SARI, respectively. Two statisticians working for CIP, Bert De Boeck and Raul Eyzaguirre, supported the data analysis. Wolfgang J. Grüneberg gave his input as an experienced sweetpotato breeder at CIP based in Peru. Jan Low was the principle investigator of the project. Hugo Campos reviewed the manuscript before final submission.

## Supporting information

Supplemental material is available online for this article.

SUPPORTING INFORMATION
